# The Global Inventor Gap: Distribution and Equality of World-Wide Inventive Effort, 1990–2010

**DOI:** 10.1371/journal.pone.0122098

**Published:** 2015-04-07

**Authors:** Hannes Toivanen, Arho Suominen

**Affiliations:** 1 VTT Technical Research Centre of Finland, Innovation, Policy & Economy, Espoo, Finland; 2 Lappeenranta University of Technology, School of Business, Lappeenranta, Finland; Universidad Veracruzana, MEXICO

## Abstract

Applying distance-to-frontier analysis, we have used 2.9 million patents and population data to assess whether the relative capacity of world countries and major regions to create new knowledge and technology has become globally more equal or less equal between 1990 and 2010. We show with the Gini coefficient that the global distribution of inventors has become more equal between major countries and regions. However, this trend has been largely due to the improved performance of only two major countries, China and India. The worst performing regions, totalling a population of almost 2 billion, are actually falling behind. Our results suggest that substantial parts of the global population have fallen further behind countries at the global frontier in their ability to create new knowledge and inventions, and that the catch-up among the least developed and middle-income countries is highly uneven, prompting questions about the nature and future of the global knowledge economy.

## Introduction

The impacts of globalization of new knowledge and technology on world development are profound, but over the last two decades two contrasting schools of thought about the nature of this impact have emerged. One, exemplified by the United Nations Millennium Goals and other [1–3], regards new knowledge and technology as critical in solving the world’s greatest challenges. This approach, often underplaying the potential of developing country innovation [4], prescribes transfer of scientific and technological solutions from rich to poor countries to address pressing development challenges.

The other school of thought, spearheaded by Manuel Castells [5] and Dani Rodrik [6], argues that since the early 1990s the nature of new technologies and the process of globalization have been amplifying the advantages of the high-skilled, networked, and rich people and nations, and exacerbating the disadvantages of low-skilled, isolated and the poor ones. The question boils down whether new knowledge and technology are narrowing or widening the gap between the”losers” and”winners” of globalization, and the debate has become polarized on the strategic viability of nations as “users” or “creators” of new knowledge and technology.

Over the last two decades, students of the role of technology in advancing global development have increasingly focused on the role of broader organizational and societal capabilities. Several studies on national innovation systems and development, departing from the definitional work of Coehen and Levinthal [7], have shown that the adoption, implementation, and use of new technologies, and their economic and social exploitation, is conditional on the initial level of scientific and technological sophistication of the nation, conceptualized as “national absorptive capacity”. [8–12] The theory foresees “threshold” quality requirements in science and technology capabilities for countries to successfully adopt new knowledge and technologies. In short, in order to use or imitate technologies, some threshold capacities in creating new knowledge are required.

Troublingly, as Fagerberg and Verspagen have argued, “the importance of innovation for economic growth has increased lately, while at the same time imitation (or diffusion) has become more demanding”. [13] Series of empirical studies have also indicated that the global technology gap between the bottom and frontier countries is indeed widening, raising further concerns that countries not catching-up sufficiently are less likely to improve their situation in the future either. Studying technology levels in export goods between 1972–2001, Kemeny [14] concluded that the technology gap between the most and least sophisticated countries increased. Studying the period between 1970 and 2000, Castellacci [15] demonstrated that less developed economies have been able to diminish the technology gap in terms of human capital and technological infrastructure, whereas the innovation gap between the rich and poor countries has increased.

Although a relatively small, albeit growing, body of evidence is showing that the ability of developing countries to catch-up with the global technology frontiers varies greatly and is dependent on a number of national features, the understanding of the extent and nature of the global technology gap is still vague. Considering the popularity of the idea to harness technology and innovation for development of the world’s poorest countries, as well as the prevalence of the two above-discussed contrasting views on the relationship between technology and global equality, it is important to produce additional evidence, analysis, and perspectives on the question. This paper casts new light on the issue by examining the spatial distribution of global inventive effort between 1990 and 2010. In particular, our aim is to assess the dynamics of increasing or narrowing the inventor gap between the global frontier and followers, and to detail the performance of different countries and regions.

The geography of inventive effort reflects a central trend of globalization and countries’ abilities to move towards the knowledge economy. It is also a suggestive indicator of countries’ comparative standings, as in a stylized postulation, the number of inventors per population in a country is indicative of its position in the global innovation ecosystem and the status of its knowledge economy. Countries with comparatively high intensity of inventors per population demonstrate strong capabilities in “new to the world” science and technology, as well as participation in international technology trade, and overall these countries are likely to be able to take advantage of globalization.

We studied 2.9 million USPTO granted patents and world population data [16] between 1990 and 2010 to estimate whether the global distribution of inventive effort is becoming more equal or less equal, as well as to detail the catch-up dynamics between different types of countries. Instead of relying on existing indicators or measures [9,17], we developed a distance-to-frontier indicator to measure *the global inventor gap*. Based on a fractional count of inventors from different countries and five-year moving averages, we estimated the inventor intensity (measured as fractional patents per population) for 50 countries with the most patents, and classified the rest of world countries in major regions (7 regions), and then we analyzed how different countries evolved relative to the defined world frontier (the US) between 1990 and 2010.

Our central objective is to cast new light on the relationship between technology and globalization, as well as to measure what type of countries (or clusters of countries) are able to move towards the global frontier in the inventor-per-population measure, which countries are able to catch-up significantly, and which countries are falling behind.

## Materials and Methods

We analyze the temporal change of patenting in selected nation states and major regions when controlled for the changes in population within the same time span. We focus on the aggregate temporal changes of patenting in countries measured against the frontier of patenting. We do this to uncover the evolution of equality in inventorship and if there are latent temporal changes in the dynamics of countries.

To obtain patent data, we use the Worldwide Patent Statistical Database (PATSTAT) April 2012 edition provided by the European Patent Office, which was installed on a local MySQL database. For the study, all United States Patent and Trademark Office (USPTO) publication kind first time granted utility patents with publication date between 1990 and 2010 (inclusive) were selected. The final data consisted of 2,870,550 granted U.S. patents.

The decision to restrict our analysis to USPTO patents is done in order to enhance the validity of our data and results, as there is a uniform standard for the patent granting process, as well as for the international standing of the patent. We do not believe that inclusion of so-called triadic patents would alter our results significantly, apart from probably leading to stronger representation of large multinational companies and leading industrial countries in our data. However, one should note that national patents would likely offer better coverage of inventive effort in developing countries and emerging economies, yet varying national practices and legal frameworks would undermine the viability of such data for international comparisons. One should also note that the different national practices and legal frameworks may also affect the propensity to apply for national patents, especially in developing countries, and thereby further undermine efforts and incentives to obtain USPTO patents.[18]

Our decision to focus exclusively on patents as a proxy of inventiveness has its well-established limitations too. Not all new knowledge, inventions, and innovations result in patents, and thereby it is likely that our analysis is unable to capture comprehensively the technological ingenuity in different societies. In particular, patents are a poor indicator of social and organizational innovations and inventions, and place priority on science and engineering. Nevertheless, once one bears in mind the usual caveats associated with patent indicators, patent data offers also one of the most reliable and accurate data sources to be used as a proxy for inventors.

To create a temporal dimension to the data, we have dated patents according to the publication date of granted patents, but we acknowledge that there are other alternatives, each having their advantages and disadvantages. The problems associated with our approach include the fact that there is a time lag from application to grant, and this time lag can be extended for foreign patent applications. To control for the time lag, we study the evolution over two decades, and use only five-year moving averages for calculations on inventor counts. One should note that the timing issue affects similarly bibliometrics, where publications are counted only by the publication year, as well as no effort is made to identify the initial date of submission to a journal or to control for multiple publications or versions of the same paper.

To estimate the relative number of inventors per country, we use a complete-normalized (fractional) counting scheme. For each patent we calculate the fractional share of inventorship by country by using the country code field for inventors. We include as inventors only real persons that have been assigned an inventor sequence number with a value of at least 1 in the PATSTAT database structure, and exclude all assignees from our assessment of country of inventors. We have applied the complete-normalized counting scheme of inventor countries as follows: Each patent has a score of one (1.00), and the country or region of residence of inventors are assigned their fractional share of the score. In practice, if a patent has four inventors, one resident in the U.S., one in Mexico and two others in Canada, the U.S. is credited a score of 0.25, Mexico 0.25 and Canada 0.5.

The definition of countries and major regions for analysis was done by selecting the 50 countries with the most inventors in 2005 for detailed study, and by assigning all remaining countries to their major geographical regions, which totaled 7. This was necessary, as patenting is a relatively concentrated phenomenon, resulting in highly skewed distributions, and the inclusion of the end tail of low-patenting countries would not have added to our analysis.

The top-50 countries from 2005 that were selected for further study were: Argentina, Australia, Austria, Belgium, Brazil, Canada, Chile, China P. Rep., Croatia, Czech Republic, Denmark, Egypt, Finland, France, Germany, Greece, Hong Kong S.A.R., Hungary, Iceland, India, Israel, Ireland, Italy, Japan, Liechtenstein, Luxembourg, Malaysia, Mexico, Netherlands, New Zealand, Norway, Philippines, Poland, Portugal, Romania, Russia, Saudi Arabia, Singapore, Slovenia, South Africa, South Korea, Spain, Sweden, Switzerland, Taiwan, Thailand, Turkey, Ukraine, United Kingdom, United States. All remaining countries were assigned to one of the following Major Regions groups (acronyms used in figures follow in parenthesis): Other Europe (O_E), Other Central America and the Caribbean (O_CAC), Other Middle East (O_ME), Other South America (O_SA), Other Oceania (O_O), Other Africa (O_AF), or Other Asia (O_A).

To assess countries relative inventive efforts, we calculate the share of (fractional) patents per 1,000 inhabitants per year. For worldwide population data, we use the United Nations [16] estimates of world population. Here, a number of uncertainties emerge. First, the break-up of the Soviet Block split and united a number of countries, and for a number of European countries the population estimates are completely reliable only from about the mid-1990s. For a two-decade study, it is nearly impossible to accommodate the re-drawing of political boundaries. Whether countries have been split or merged, they are listed in our analysis independently if they are one of the 50 leading countries obtaining U.S. patents in 2005. This affects some countries more than others, and the Czech Republic, Slovenia, Germany, and East Germany are cases in point. However, we do not believe this issue has a profound effect on our fundamental observations and claims.

Secondly, we have modified some of the regional groupings of the United Nations data. For example, Cyprus and Turkey have been moved to region Europe, because of their access to the research and development and other programs of the European Union. Additionally, the Middle-East Region was created, and other minor changes to the UN hierarchy have been done as well. Finally, the UN data omits population data for Taiwan, and this was obtained from the National Statistics of Taiwan.[16,19]

Assigning credit for each patent yearly according to the before-mentioned counting scheme and calculating the sum of assigned credits yearly yields a continuous variable FP_ct_, representing the sum of fractional patent shares for a country or region c at a given year t. To control the FP_ct_ variable by the population of a country we create P_ct_ variable—the population of a given country or region c at a given year t. Finally, for both variables, we control for possible yearly variations in the data by calculating a simple five-year moving average for both variables, creating the variables SMAFP_cy_ and SMAP_cy_, where c is a country’s region and y is the starting year for the moving average.

To assess the distance to frontier between low- and high-intensity inventor countries, we have defined the U.S. as the global frontier, and subsequently calculated the evolution of the distance of other countries and regions yearly to the U.S. The U.S. represents a natural invention frontier because U.S. residents have a higher propensity to obtain U.S. patents than foreigners, and the country has a high over-all international patenting rate. Secondly, inventors resident in other countries must make serious internationalization efforts and investments to obtain U.S. patents, thus an increase or decrease in a country’s U.S. patents can be interpreted as a signal of participation in the global high-technology market. We define a catch-up index for each country by,

$$C_{cy}=\frac{SMAFP_{cy}/SMAP_{cy}}{SMAFP_{USy}/SMAP_{USy}},$$

where *SMAFP*_*USy*_ and *SMAP*_*USy*_ are the moving average value for the US. To calculate the yearly change in the distance to the frontier, we further calculate catch-up ratio for each country for year y+1as,

CRcy+1=CcyCcy+1.

Thus, a catch-up ratio smaller than 1 indicates that a country is falling behind the US, a catch-up ratio of one indicates that its distance remains the same relative to the US, and a catch-up ratio higher than one indicates that it is catching up to the US.

To measure the inequality within the distribution of SMAFP_cy_ we calculated the Gini coefficient. We weighted the Gini coefficient with the population data using the SMAP_cy_ variable. The calculations have been done in the R statistical program in the package ‘reldist’. The value of the coefficient is from 0 to 1, where 1 is defined as maximal inequality.

Finally, to identify countries that have the greatest impact on the re-allocation of the distribution of global inventive effort, we cluster countries and regions based on two time cohorts, 1990–1995 and 2005–2010. We used a log transformation for the variable SMAFP_cy_ and, to uncover the emergence of clusters of countries in the data, we run a hierarchical clustering analysis. The log transformation was carried out to show the skewness of the SMAP_cy_ distributions_._ Using the R package ‘pvclust’ we assess the uncertainty in hierarchical cluster analysis by running a hierarchical cluster analysis with a multiscale bootstrap with a number of bootstrap 10,000, using the average method and the Euclidean dissimilarity matrix. The method calculates p-values via multiscale bootstrap resampling for each cluster in hierarchical clustering. The p-value of a given cluster is between 0 and 1, indicating the strength by which the cluster is supported by the data. For a cluster with approximately unbiased p-value > 0.95, we can reject the hypothesis that the cluster does not exist.

## Results

Patenting is known to be highly concentrated, and descriptive statistics suggest that the overall distribution of inventors per world population has changed between 1990 and 2010 relatively little. Whereas the eight countries with the most inventors between 1990–1995 accounted for 93.42% of all patents and 11.93% of the world population, in 2005–2010 the eight countries with most patents accounted 90.93% and 10.86%, respectively.

**Fig 1:**
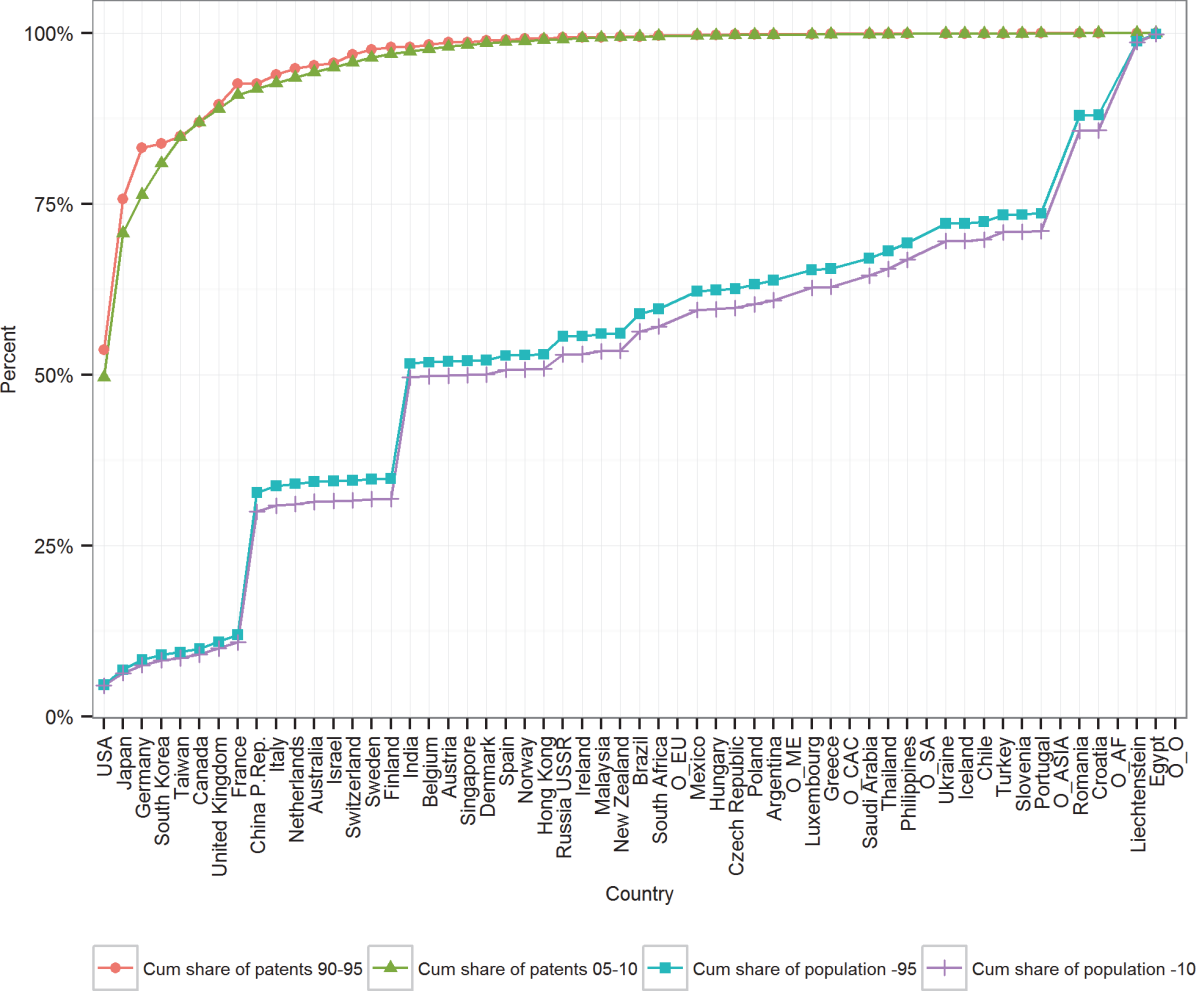
The cumulative share of fractional inventors and world population by 57 major countries and regions 1990–1995 and 2005–2010. Countries and regions on the horizontal axis are ranked in descending order of count of fractional patents in 2005–2010. The vertical axis shows the cumulative share from all granted USPTO patents in 1990–1995 and 2005–2010. Countries are represented by the two letter abbreviations and Major Regions as explained above.

Fig 1 illustrates two other major developments in the distribution of global inventive effort: First, the global inventor frontier has become slightly more flat, demonstrating an intensified competition between high-intensity inventor nations. Secondly, population growth and increased inventive effort in China and India have tipped the distribution of global population slightly towards the inventive frontier. (Fig 1)

Troubling results for global efforts for development are presented in Table 1, which shows the catch-up ratio as percentage change for the distance of countries and regions relative to the U.S., the defined global inventor frontier. While one would expect that the relative position of high-performing innovation countries would decline most, it is the medium range region Other South America (excluding Argentina, Brazil, and Chile) whose relative distance to the U.S has increased most: 54% percent between 1990 and 2010. The country falling behind the global frontier at the second highest rate is South Africa, whose relative distance to the U.S. has increased 47%. Of other major developing regions and countries falling further behind, the distance of Other Africa to the U.S. has increased 33%, and the respective figure for Other Central America and Caribbean is 4%.

**Table 1:** Global patent catch-up ratio for countries and major regions 1990–2010.

		****Comparing periods****				
****Rank****	****Country or Major Region****	****1990–1995 / 1995–2000****	****1995–2000 / 2000–2005****	****2000–2005 / 2005–2010****	****1990–1995 / 2005–2010****	****Population 2010 (1000')****
1	**China P.Rep.**	21%	231%	302%	1505%	1318194
2	**India**	95%	194%	124%	1183%	1224614
3	**Malaysia**	66%	121%	133%	756%	28401
4	**South Korea**	175%	38%	93%	635%	48184
5	**Singapore**	144%	145%	7%	540%	5086
6	**Croatia**	315%	0%	50%	522%	4403
7	**Ukraine**	332%	36%	5%	514%	45448
8	**Slovenia**	301%	42%	3%	486%	2030
9	**Romania**	66%	58%	117%	473%	21486
10	**Turkey**	59%	133%	41%	420%	72752
11	**Other Oceania**	677%	-36%	-8%	356%	9956
12	**Thailand**	128%	55%	6%	275%	69122
13	**Taiwan**	86%	61%	24%	270%	23141
14	**Philippines**	17%	114%	39%	246%	93261
15	**Iceland**	33%	109%	10%	205%	320
16	**Poland**	41%	3%	98%	187%	38277
17	**Portugal**	15%	73%	32%	162%	10676
18	**Other Middle East**	27%	53%	23%	138%	124378
19	**Hong Kong**	39%	48%	6%	119%	7053
20	**Ireland**	20%	47%	21%	114%	4470
21	**Greece**	23%	9%	50%	101%	11359
22	**Egypt**	-2%	75%	15%	96%	81121
23	**Israel**	24%	31%	19%	93%	7418
24	**Finland**	23%	32%	17%	89%	5365
25	**Saudi Arabia**	40%	-2%	37%	89%	27448
26	**New Zealand**	50%	25%	-3%	83%	4368
27	**Australia**	0%	25%	44%	79%	22268
28	**Chile**	7%	6%	56%	78%	17114
29	**Other Europe**	-36%	30%	95%	60%	53495
30	**Czech Republic**	-17%	33%	39%	54%	10493
31	**Denmark**	39%	13%	-2%	54%	5550
32	**Norway**	16%	20%	8%	50%	4883
33	**Spain**	11%	17%	9%	41%	46077
34	**Russia**	14%	12%	8%	39%	142958
35	**Brazil**	2%	13%	7%	24%	194946
36	**Belgium**	30%	0%	-7%	20%	10712
37	**Japan**	-3%	10%	11%	18%	126536
38	**Canada**	2%	5%	6%	14%	34017
39	**Sweden**	21%	17%	-21%	11%	9380
40	**Mexico**	9%	23%	-20%	8%	113423
41	**Austria**	-9%	14%	-1%	4%	8394
42	**Argentina**	23%	4%	-19%	3%	40412
43	**USA**	0%	0%	0%	0%	310384
44	**Netherlands**	-11%	6%	6%	-1%	16613
45	**Germany**	-12%	16%	-5%	-3%	82302
46	**Other Central America and Caribbean**	19%	-4%	-16%	-4%	84169
47	**United Kingdom**	-6%	3%	-3%	-6%	62036
48	**Other Asia**	-8%	7%	-6%	-8%	988665
49	**Italy**	-11%	3%	-11%	-18%	60551
50	**Luxembourg**	-31%	43%	-17%	-18%	507
51	**France**	-12%	-4%	-8%	-22%	62787
52	**Other Africa**	-52%	33%	5%	-33%	890981
53	**Switzerland**	-25%	-7%	-7%	-35%	7664
54	**Hungary**	-52%	12%	18%	-37%	9984
55	**Liechtenstein**	-23%	0%	-25%	-42%	36
56	**South Africa**	-25%	-20%	-12%	-47%	50133
57	**Other South America**	-14%	-27%	-31%	-56%	140082

Catch-up ratio is based on a moving five-year average of fractional patents per population. Countries and major regions are ranked in order of their catch-up ratio in 1990–2010. Additionally, the table details the catch-up ratio for consecutive five-year periods between 1990 and 2010, detailing the varying speeds of catch-up, stagnation, and falling behind of individual countries and major regions. Sources: Authors’ own data; [16,19]

**Fig 2:**
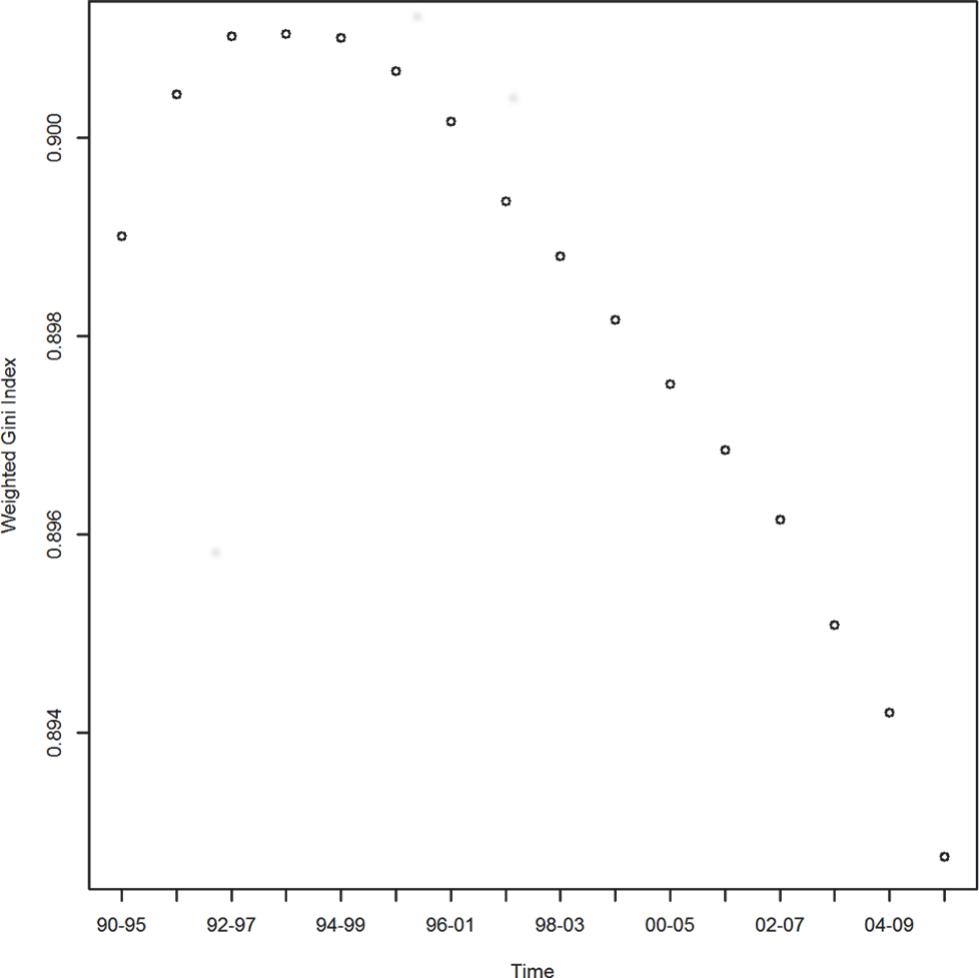
Gini coefficient for global inventive effort 1990–2010. Gini coefficient calculated for fractional patents and five-year moving averages weighted with variable SMAFP_cy_ distributions. This plot presents the change in equality of patenting, where a decreasing value implies a more equal distribution of patenting. Each point in the figure represents the population weighted Gini coefficient at a given five-year moving average. The Gini coefficient increases initially, but since mid-1990s declines steadily until 2010.

To go beyond descriptive statistics and to assess whether the global inventive effort has become more equal or less equal, we have calculated the Gini coefficient. Fig 2 shows a moderate increase in the Gini coefficient. The first half of the 1990s, indicates increased global inequality, but it declines steadily after that until 2010, suggesting that overall the equality in patenting is increasing. However, to understand more fully this perceived increase in equality, it is necessary to analyze the evolution of global inventive effort on the country level.

**Fig 3:**
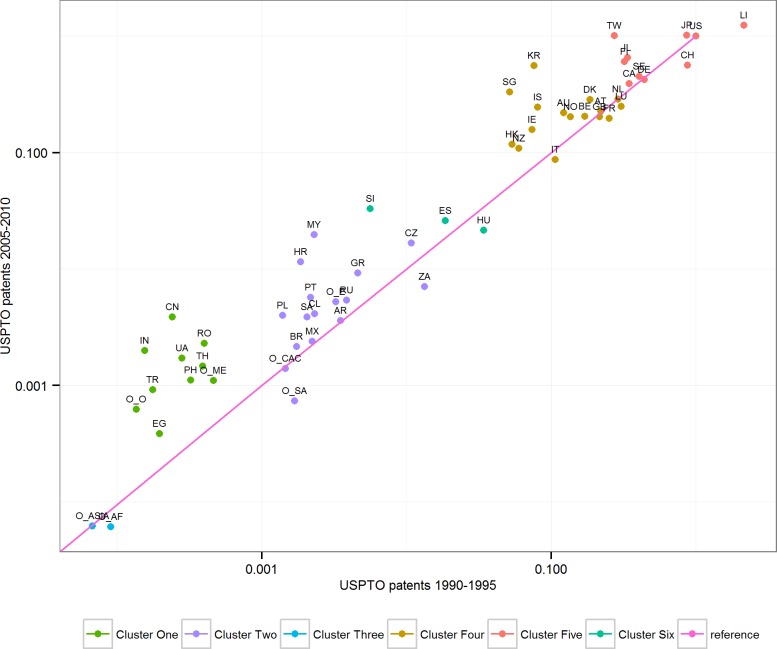
Hierarchical clustering analysis of temporal change in catch-up. The figure plots countries and aggregate regions based on two time slices, 1990–1995 and 2005–2010. A hierarchical cluster model was fitted to create a cluster differentiated by the legend. Countries are represented by the two letter abbreviations and Major Regions as explained above.

In order to identify what countries or major regions, as well as clusters of them, appear to be able to catch-up with the global frontier, and which appear to catch-up only a little or decline, we visualize the evolution of the global inventor gap in Fig 3. It plots each of the 57 countries or major regions on the horizontal axis depending on its fractional count of USPTO patents in 1990–1995 per population, whereas the vertical axis provides the same value for the years 2005–2010. Both axes are logarithmic.

**Fig 4:**
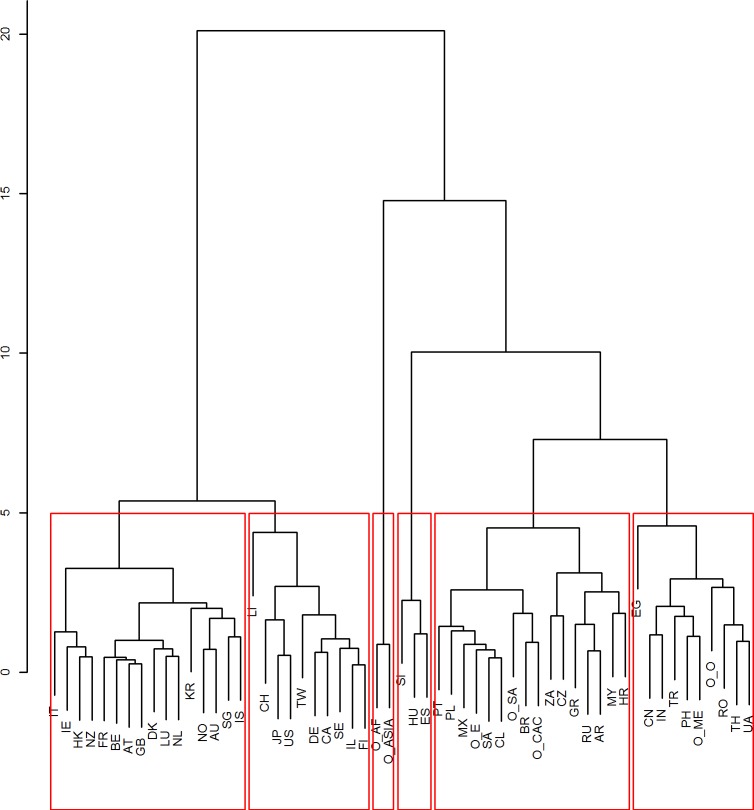
Statistical support for the clustering of countries and major regions. The dendogram graphically shows the clusters which are statistically supported. The AU values, given at the left of each split, are Approximately Unbiased p-values computed by multiscale bootstrap resampling. The BP (Bootstrap Probability) values at the right of each split are p-values computed by normal bootstrap resampling. The AU values are used to approximate the existence of a cluster and there is statistical support for clusters with AU values higher or equal to 95. Two rectangle boxes surround statistically significant clusters with an inclusion relation for which the largest cluster in surrounded with a box. Countries are represented by the two letter abbreviations and Major Regions as explained above.

**Fig 5:**
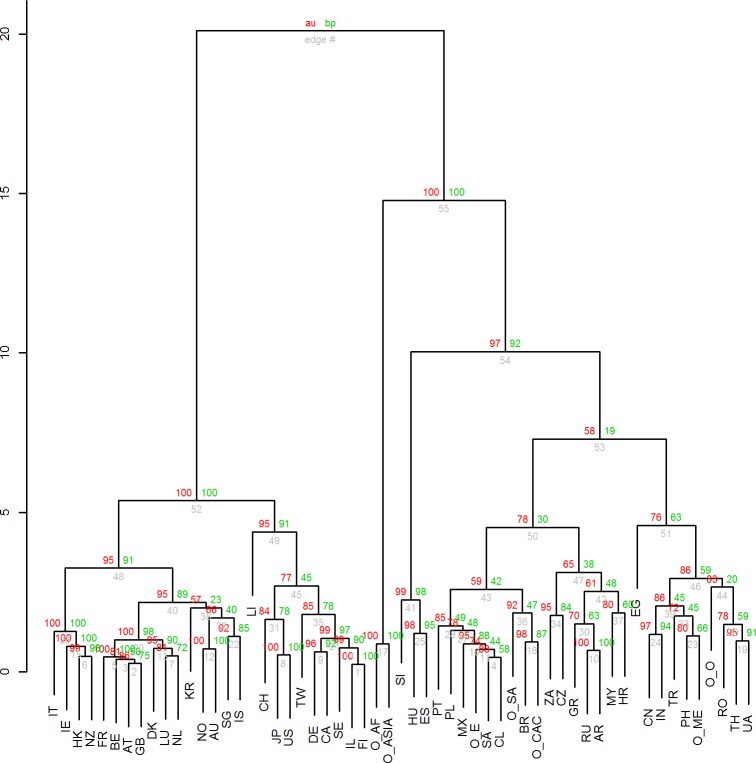
Dendogram for qualitative clustering of countries and major regions. The figure illustrates the qualitatively selected clusters used in Fig 3. The two clusters on the far right lack statistical relevance and are separated for illustrative purposes. For statistically significant clusters refer to S1 Table. Countries are represented by the two letter abbreviations and Major Regions as explained above.

Embedded in Fig 3 is the cluster analysis results. We evaluated the number of clusters through the unbiased p-value > 0.95, rejecting the hypothesis that the cluster does not exist. Seen in Fig 4, a number of options in selecting clusters emerge. Through evaluating Fig 3 and Fig 4, we selected six cluster results seen in Fig 5. Of these six clusters, four have statistical support. Fig 5 also depicts that two clusters have limited statistical support (clusters on the far right), as only partial clusters among countries, such as the clusters created by China and India, are statistically supported. The selection of six clusters is qualitative and allows for a more detailed discussion than merging the three clusters on the far right of the dendogram in Fig 5. All of the clusters are also given in S1 Table, which details statistical values for them too.

To identify countries and major regions performances vis-á-vis the U.S. in 1990–2010 we draw a line across the graph. The greater distance countries and regions are above this line, the more they improve their inventive performance, or catch-up with the global inventor frontier. Likewise, a position under the line implies decline, and falling further behind the global inventor frontier. (Fig 3)

Combined, this analysis shows that the major inventor countries and regions of the world can effectively be divided into six clusters. Clusters four and five in Fig 3 comprise the global inventor frontier. Clusters one, two, and six include a broad-range of middle-tier countries and regions, whereas cluster three is comprised of bottom countries and regions.

To understand how the global inventor gap evolves during 1990–2010, it is essential to analyze the clusters of Fig 3. Countries in cluster five and four show a persistent ability to improve or maintain their positions, as only a few countries decline, and in that case only slightly.

Countries and regions in clusters two, five and six show diverging performance. Hungary, South Africa, Other South America, and Other Central America and Caribbean show decline. Indeed, the distance of South Africa and Other South America to the global frontier increases the most of all countries and regions in the data. The relative position of Brazil, Mexico, and Argentina remains practically unchanged. A number of countries accomplish significant improvements, catching up with the global frontier. These countries include Malaysia, Slovenia and Croatia.

Cluster one consists of countries and regions with an initially low intensity of inventors, but demonstrates relatively good rates of catch-up. Within this cluster are also the two best performing countries in the data, China and India, but the cluster includes also Romania, Ukraine, Thailand, Philippines, Other Middle East, Turkey, Other Oceania, as well as Egypt. Cluster three consists of global bottom regions: Other Asia and Other Africa. While the relative position of Other Asia remains practically unchanged, the distance of Other Africa from the global inventor frontier increases.

## Discussion and Conclusions

The global inventive effort, when measured on national and regional levels, has become more equal between 1990 and 2010. However, differences in the performance of countries and major regions are significant, and only two countries, China and India, account for a significant share of the increased global equality in inventive effort. This contrasting performance is best illustrated by the countries and regions with the lowest levels of inventors (clusters one and three in Fig 3), where cluster one shows significant catch-up between 1990 and 2010, but the two bottom regions in cluster three, Other Africa and Other Asia, have fallen further behind the global frontier.

Similarly, in the middle-performing clusters (two and six) of countries (Fig 3), one can easily distinguish between significant catch-up (e.g. Malaysia), stagnant countries and regions (Argentina, Brazil, Other Central America and Caribbean), as well as countries and regions (Other South America and South Africa) that have declined.

Countries and major regions at or nearby the global inventor frontier (clusters four and five in Fig 3) demonstrate increased competitiveness. The best improvers in this group are South Korea, Singapore and Taiwan, and there are practically no countries with significant levels of decline, with the possible exceptions of Switzerland and Liechtenstein.

Thus, we can conclude that the improvement to invent between 1990 and 2010 varies relatively greatly among catch-up countries (clusters one, two, three, six), underlining how heterogeneous the catch-up phenomena is. In contrast, the club of high-intensity inventor countries near or at the global frontier shows little divergence, apart from the few countries that catch-up significantly, and can be characterized as relatively homogenous.

Our results complicate assumptions about straightforward relationships between existing organizational capabilities and the future scientific and technological performance of countries discussed in our introduction. Countries and major regions with relatively similar starting points in our analysis can have highly diverging developments. A comparison of pairs of Czech Republic-South Africa, Central America and Caribbean-Other South America-Poland, or Mexico-Portugal-Chile, in Fig 3, shows differing performance despite highly similar starting conditions. However, if we restrict analysis to the distance between the most inventive cluster five and the bottom cluster three in Fig 3, it is obvious that the inventor gap between them grew during 1990–2010.

Analysis of catching-up and falling behind in production of scientific publications has indicated broadly similar regional trends in terms of stagnation and catch-up, yet some important differences persist between analysis based on scientific publications and patents. Whereas Asia, especially China and India, figure strongly as an emerging region both in science and patents, South America demonstrates strong catch-up only in science and was an underperformer in our study. [20] Likewise, the rise of African science in the 2000s is not reflected in our analysis. [21].

While our results confirm that there is indeed increased equality in the global inventive effort when measured on the level of countries and major regions, this perceived improvement itself is highly concentrated phenomenon. Although our analysis does not extend to intra-country or intra-regional equality of inventive effort, it is worthwhile to speculate about it, as it likely bears significantly upon the nature of the catch-up process in countries and regions with large populations. It would be important for further research to investigate the distribution and equality of inventive effort within, say, China and India.

## Supporting Information

S1 Dataset Dataset parts 1 and 2. Included in the zip-file is a read_me.txt, which details the data and user instructions. The zip-file contains following data-files: **Datafile dataset_part1_uspto_grants_1990-2000.csv.** This file contains USPTO granted patents with a publication date during 1990–2000 and used in the study. **Datafile dataset_part2_uspto_grants_2001-2010.csv.** This file contains USPTO granted patents with a publication date during 2001–2010 and used in the study.
(ZIP)

S1 Table Statistically significant clusters. The table shows all of the statistically significant clusters in the hierarchical cluster analysis. In total, 24 clusters are presented with Approximately Unbiased p-values (AU), Bootstrap Probability values and Standard Error values for both AU (SE AU) and BP (SE BP). Countries are represented by the two letter abbreviations and Major Regions as explained above.
(DOCX)
