# Supplementary material for: The Global Inventor Gap: Distribution and Equality of World-Wide Inventive Effort, 1990–2010
Source: PLoS One. 2015 Apr 7;10(4):e0122098. doi: 10.1371/journal.pone.0122098 (PMC4388728; doi:10.1371/journal.pone.0122098)
Supplement: S1 Table — The table shows all of the statistically significant clusters in the hierarchical cluster analysis. In total, 24 clusters are presented with Approximately Unbiased p-values (AU), Bootstrap Probability values and Standard Error values for both AU (SE AU) and BP (SE BP). Countries are represented by the two letter abbreviations and Major Regions as explained above. (DOCX) [file pone.0122098.s002.docx]

| **Nro** | **Countries in cluster** | **AU** | **BP** | **SE AU** | **SE BP** |
| --- | --- | --- | --- | --- | --- |
| 1 | IL; FI | 1 | 1 | 0 | 0 |
| 2 | AT; BE; FR; GB | 1 | 0.998 | 0 | 0 |
| 3 | HK; NZ | 0.987 | 0.977 | 0.002 | 0.001 |
| 4 | JP; US | 1 | 1 | 0 | 0 |
| 5 | DE; CA | 0.962 | 0.924 | 0.003 | 0.001 |
| 6 | RU; AR | 1 | 1 | 0 | 0 |
| 7 | NO; AU | 0.998 | 0.995 | 0 | 0 |
| 8 | IL; FI; SE | 0.986 | 0.902 | 0.001 | 0.001 |
| 9 | HK; IE; NZ | 1 | 0.995 | 0 | 0 |
| 10 | O_AF; O_ASIA | 1 | 1 | 0 | 0 |
| 11 | BR; O_CAC | 0.982 | 0.872 | 0.001 | 0.001 |
| 12 | TH; UA | 0.954 | 0.915 | 0.003 | 0.001 |
| 13 | AT; BE; DK; FR; LU; NL; GB | 0.998 | 0.984 | 0 | 0 |
| 14 | IL; FI; DE; SE; CA | 0.993 | 0.973 | 0.001 | 0.001 |
| 15 | CN; IN | 0.969 | 0.942 | 0.003 | 0.001 |
| 16 | HU; ES | 0.976 | 0.95 | 0.002 | 0.001 |
| 17 | HK; IE; IT; NZ | 0.999 | 0.999 | 0 | 0 |
| 18 | SG; KR; AT; BE; DK; FR; IS; LU; NL; NO; GB; AU | 0.951 | 0.89 | 0.003 | 0.001 |
| 19 | HU; SI; ES | 0.986 | 0.976 | 0.002 | 0.001 |
| 20 | HK; SG; KR; AT; BE; DK; FR; IS; IE; IT; LU; NL; NO; GB; AU; NZ | 0.951 | 0.906 | 0.003 | 0.001 |
| 21 | JP; TW; IL; FI; DE; LI; SE; CH; CA; US | 0.951 | 0.906 | 0.003 | 0.001 |
| 22 | HK; JP; SG; KR; TW; IL; AT; BE; DK; FI; FR; DE; IS; IE; IT; LI; LU; NL; NO; SE; CH; GB; CA; US; AU; NZ | 1 | 1 | 0 | 0 |
| 23 | EG; ZA; CN; IN; MY; PH; TH; SA; O_ME; HR; CZ; GR; HU; PL; PT; RO; RU; SI; ES; TR; UA; O_E; O_O; AR; BR; CL; O_SA; MX; O_CAC | 0.969 | 0.92 | 0.002 | 0.001 |
| 24 | EG; ZA; O_AF; CN; IN; MY; PH; TH; O_ASIA; SA; O_ME; HR; CZ; GR; HU; PL; PT; RO; RU; SI; ES; TR; UA; O_E; O_O; AR; BR; CL; O_SA; MX; O_CAC | 1 | 1 | 0 | 0 |

**Table S1. Statistically significant clusters.** The table shows all of the statistically significant clusters in the hierarchical cluster analysis. In total, 24 clusters are presented with Approximately Unbiased p-values (AU), Bootstrap Probability values and Standard Error values for both AU (SE AU) and BP (SE BP). Countries are represented by the two letter abbreviations and Major Regions as explained above.
